# Multisensory Integration: Is Medial Prefrontal Cortex Signaling Relevant for the Treatment of Higher-Order Visual Dysfunctions?

**DOI:** 10.3389/fnmol.2021.806376

**Published:** 2022-01-17

**Authors:** Miguel Skirzewski, Stéphane Molotchnikoff, Luis F. Hernandez, José Fernando Maya-Vetencourt

**Affiliations:** ^1^Rodent Cognition Research and Innovation Core, University of Western Ontario, London, ON, Canada; ^2^Département de Sciences Biologiques, Université de Montréal, Montreal, QC, Canada; ^3^Département de Génie Electrique et Génie Informatique, Université de Sherbrooke, Sherbrooke, QC, Canada; ^4^Knoebel Institute for Healthy Aging, University of Denver, Denver, CO, United States; ^5^Department of Biology, University of Pisa, Pisa, Italy; ^6^Centre for Synaptic Neuroscience, Istituto Italiano di Tecnologia (IIT), Genova, Italy

**Keywords:** visual cortex, prefrontal cortex, multisensory integration, blindness, higher-order visual impairments, CVI, rescue of vision, environmental enrichment

## Abstract

In the mammalian brain, information processing in sensory modalities and global mechanisms of multisensory integration facilitate perception. Emerging experimental evidence suggests that the contribution of multisensory integration to sensory perception is far more complex than previously expected. Here we revise how associative areas such as the prefrontal cortex, which receive and integrate inputs from diverse sensory modalities, can affect information processing in unisensory systems *via* processes of down-stream signaling. We focus our attention on the influence of the medial prefrontal cortex on the processing of information in the visual system and whether this phenomenon can be clinically used to treat higher-order visual dysfunctions. We propose that non-invasive and multisensory stimulation strategies such as environmental enrichment and/or attention-related tasks could be of clinical relevance to fight cerebral visual impairment.

## Introduction

The integration of sensory information underlies a coherent perception of the environment. Initially, multisensory integration was thought to take place solely in dedicated brain regions such as the association cortices (Ghazanfar and Schroeder, 2006; Driver and Noesselt, 2008) or the superior colliculus (Stein and Arigbede, 1972; Wallace et al., 1996), which receive converging inputs from multiple primary unisensory areas. There is evidence, however, that multisensory interactions also occur in primary sensory systems. This is epitomized by the existence of non-visual influences on visual cortical neurons (Fishman and Michael, 1973; Bulkin and Groh, 2006), non-auditory influences in the auditory cortex (Sams et al., 1991; Bourguignon et al., 2020), and non-somatosensory influences on somatosensory cells (Jabbur et al., 1971; Zarzecki et al., 1983). These early findings suggest that it may be possible to modulate unisensory perception by stimulating different sensory modalities. In light of this, multisensory stimulation strategies that activate high-order associative areas such as the prefrontal cortex (PFC) could be of clinical relevance to fight different neurological disorders.

The nervous system relies on processes of sensory integration in association cortices to generate behavioral responses to changing environmental conditions. In primates, for instance, physiological mechanisms that subserve decision-making include the synchronized activity of the amygdala and PFC. When monkeys decide whether a conspecific should receive rewards, neuronal activity synchronization between the basolateral amygdala (BLA) and the anterior cingulate gyrus (ACCg) in the PFC is enhanced in the beta and gamma frequency bands but not in cases of anti-social decisions (Dal Monte et al., 2020). This points toward a facilitating role for inter-regional synchrony in primate social behavior. Anatomical projections between the ACCg and BLA have been described (Klavir et al., 2013) and studies in primates using a reward-allocation task demonstrated that there are neurons in the ACCg that encode reward allocations to other conspecifics (Chang et al., 2013).

Another example of multisensory integration that subserves behavior is represented by the observation that amygdala projections to the medial PFC (mPFC) seem to regulate anxiety. Behavioral findings using the elevated plus maze and the open field test revealed that the optogenetic activation of BLA projections to the mPFC in freely moving mice increases anxiety-related behavior whereas the optogenetic inhibition of BLA inputs decreases it (Felix-Ortiz et al., 2016). Consistently, hyperactivity of the BLA in humans (Rauch et al., 2003) and rodents (Rosenkranz et al., 2010) has been reported in anxiety disorders. In addition, the mPFC shares reciprocal connections with the BLA (Hoover and Vertes, 2007), and the enhanced neuronal activity in the mPFC correlates with heightened anxiety-related behavior (Bi et al., 2013). Of note, electrophysiological recordings in mice observing a conspecific show that anterior cingulate cortex (ACC) inputs to the BLA are necessary for observational learning. Interestingly, optogenetically inhibiting ACC-BLA projections prevents this behavioral phenomenon in rodents (Allsop et al., 2018).

### Multisensory Integration in the Brain

**Figure 1:**
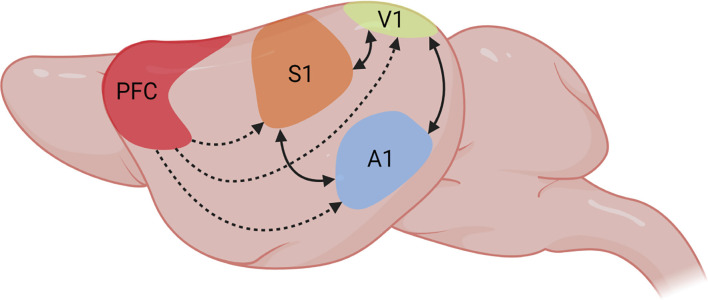
Multisensory integration in primary sensory areas. Representation of multisensory integration processes between PFC and diverse primary sensory areas in the rodent brain. The PFC has been proposed as the source of top-down attention signals that modulate information processing in primary unisensory areas in favor of the attended features (dashed arrows). Continuous arrows represent bidirectional modulation of information processing between diverse sensory modalities. Figure created with BioRender.com.

Phenomena of brain regions cross-talk have been brought to light by different electrophysiological and imaging studies, which have proved neuronal responses in the primary visual cortex (V1) after single sound stimulation in mice (Iurilli et al., 2012), cats (Morrell, 1972), primates (Rockland and Ojima, 2003; Clavagnier et al., 2004), and humans (Martuzzi et al., 2007; Vetter et al., 2014). This notion has been confirmed by the identification of different anatomical pathways that could mediate auditory responses in V1 (Falchier et al., 2002; Komura et al., 2005; Figure 1). It remains an open question whether V1 responses, under pathological conditions, can be modified after long-term sound stimulation. Recent experimental findings in cats demonstrated that the prolonged presentation of an auditory stimulus recalibrates the orientation selectivity of visual cortical neurons. Extracellular recordings of neuronal activity in anesthetized cats revealed that orientation tuning curves of neurons in both supra-granular and infra-granular layers of V1 significantly shift in response to a 12 min-long presentation of acoustic stimuli (Chanauria et al., 2019). Accordingly, pure tones improve the representation of orientation and direction of visual stimuli in mice (McClure and Polack, 2019). All these findings show that V1 pyramidal neurons dynamically integrate features of sound. Consistently, orientation selectivity in superficial layers of V1 in mice is sharpened in the presence of a sound or after optogenetic activation of A1 areas (Ibrahim et al., 2016). Layer 1 neurons in V1 are strongly activated by sounds whereas layers 2/3 neurons activity is inhibited. Of note, suppressing layer 1 activity reduces the cross-modal phenomenon in layers 2/3. This indicates that intracortical inhibitory/disinhibitory processes in superficial layers of V1 modulate cross-modal A1 signals that arrive at and modulate V1 activity. Imaging studies in humans show that there might be salient locations within V1 that respond to other sensory-specific cross-modal inputs (Liang et al., 2013). Thus, it seems reasonable to hypothesize that under appropriate conditions it might be possible to permanently adjust the functional properties of visual cortical neurons by long-term sound exposure. To what extent this can be achieved in the impaired V1 and how much time this phenomenon prevails remains to be investigated.

Multisensory phenomena have also been reported in the primary auditory cortex (A1) after V1 stimulation. Recordings of neuronal activity in A1 of alert monkeys exposed to audio-visual stimuli revealed that reliability of neuronal responses increases after the presentation of bimodal stimuli, as compared to A1 activity when the visual stimulus does not match sounds (Kayser et al., 2010). This suggests that multisensory influences boost information processing in primary unisensory areas. Electrophysiological recordings of A1 multiunit activity in awake macaques have also shown somatosensory-auditory interactions during sensory processing. Signals in the primary somatosensory cortex (S1) seem to modulate the phase of neuronal oscillations in A1 to ensure the arrival of auditory inputs in a high excitability state thus amplifying neuronal responses (Lakatos et al., 2007). Instead, A1 inputs arriving during a low-excitability phase are normally suppressed. This highlights an interesting role for neuronal oscillations in information processing of sensory areas. On the other hand, extracellular recordings in the barrel field of rats revealed that simultaneous light flashing and whisker deflection enhances S1 responses and resets the phase of neuronal networks oscillations, as compared to S1 activation alone (Sieben et al., 2013). The pharmacological silencing of V1 decreases but does not abolish cross-modal effects on S1 oscillatory activity. Consistently, there is anatomical connectivity between these two sensory areas. V1 inputs seem to impact S1 processing by modifying neuronal networks oscillations *via* corticocortical projections and subcortical feedforward interactions. There is also evidence that the reliability of sensory signals modulates processes of sensory integration. Auditory stimuli influence tactile perception whereas touch biases auditory perception. Decreasing the intensity of signals reduces the influence of audition on touch while increasing the effects of touch in audition (Bresciani and Ernst, 2007).

### Neuromodulators and Neuronal Networks Interactions

In addition to intracortical inhibitory and excitatory processes, neuromodulators such as acetylcholine (ACh), serotonin (5-HT), norepinephrine (NE), and dopamine (DA) also play a key role in integrating multisensory information, as they are involved in the regulation of oscillatory network activity. Arousal systems in the brain subserve the generation of cortical activation and sensory-motor responsiveness. They work in parallel and are grossly redundant, although differentiated in their specific roles and behavioral responses they orchestrate. For instance, basal forebrain ACh neurons give rise to ascending projections that parallel those of the reticular formation regulating wakefulness and arousal, and they promote cortical activation during waking (Steriade et al., 1991) and rapid eye movements during sleep (El Mansari et al., 1989). As to 5-HT raphe neurons, early studies demonstrated that stimulation of midbrain raphe nuclei positively correlates with behavioral arousal (Jacogs et al., 1973), and they are implicated in executive functions, motivation, learning, and memory (Meneses and Liy-Salmeron, 2012). NE neurons in locus coeruleus also have the capacity to influence different cortical areas in virtue of a diffused innervation of the entire brain (Jones and Moore, 1977). Such signals support aroused waking states by activating the neocortex and hippocampus (Berridge and Foote, 1991), and are involved in cognitive functions such as attention and working memory (Aston-Jones and Cohen, 2005). Finally, mesencephalic dopaminergic neurons from the substantia nigra pars compacta (SNc) and ventral tegmental area (VTA) constitute, respectively, the mesostriatal and mesocorticolimbic systems (Bjorklund and Dunnett, 2007). DA neurons in the SNc regulate voluntary movements and postural reflexes while VTA neurons are involved in the regulation of goal-directed behaviors, reward, attention, and cognitive processing.

Previous reports have suggested that network circuitry mechanisms mediated by the actions of diverse neuromodulatory systems across mPFC and subcortical structures, can collectively trigger further functional modifications of neuronal circuitries in primary sensory areas. For instance, stimulation of basal forebrain cholinergic neurons during spatial learning and working memory tasks robustly increase neuronal responses, cue detection ability, and long-term facilitation in A1, with a clear expansion of the cortical area that represents the paired frequency (Bentley et al., 2004; Keuroghlian and Knudsen, 2007; Bauer et al., 2012). There is also evidence that 5-HT in the visual system may serve in mechanisms of attention, arousal, and motivation. It has been reported that 5-HT exerts a strong modulation of gamma oscillations in the PFC of rats *via* 5-HT_1A_ and 5-HT_2A_ receptors (Puig et al., 2010), suggesting a potential role in the control of neuronal network activity in V1 depending on the animal’s behavioral and/or motivational context (Seillier et al., 2017; Garner and Keller, 2021). A recent study in humans highlighted that NE modulates the activity and sensory perception in V1 (Gelbard-Sagiv et al., 2018). In addition, NE seems to be an important modulator of synchronic oscillatory activity in PFC that underlies cognitive function (Dalley et al., 2004). It is still an open question whether NE-evoked oscillatory activity in PFC can directly or indirectly influence network activity in V1. Lastly, studies in rodents and monkeys have also identified DA neurons in SNc/VTA that make key contributions to associative learning and decision-making, at least in part, by encoding reward prediction errors (Schultz, 2016). Reports in rodents (Shuler and Bear, 2006), monkeys (Arsenault et al., 2013; Stanisor et al., 2013), and humans (Serences and Saproo, 2010; Vickery et al., 2011) suggest that DA reward modulates features representation in V1 while encoding reward uncertainty in the mPFC (Starkweather et al., 2018). This suggests that the interaction of network circuitry mechanisms across cortical structures (including the mPFC and V1) is influenced by a complex and heterogeneous neuromodulatory signaling regulating neuronal excitability.

### Top-Down Effects of PFC Signals on the Visual Pathway

The generation of oneiric images during sleep illustrates the influence of PFC signals on visual cortical areas. While imaging experiments in humans have shown that the interaction between the PFC and temporal-parietal association regions play a key role in dream experience (Muzur et al., 2002; Eichenlaub et al., 2014; Baird et al., 2018), different studies have demonstrated that focal lesions in the mPFC, unilateral or bilateral, are consistently associated with a marked decrease or total cessation of dreaming during sleep, respectively (Solms, 2000). Likewise, it has been reported that sleep promotes V1 plasticity in cats during early development (Frank et al., 2001), such phenomenon being dependent on endogenous cortical excitability in the sleeping brain (Jha et al., 2005). This suggests that PFC activity, in concert with other brain areas, modifies synaptic circuitries in V1. Consistently, sleep facilitates synaptic plasticity-dependent processes of visual discrimination learning (Stickgold et al., 2000). It is no wonder, then, that rapid eye movement (REM) phases of sleep in which dreaming largely occurs promote experience-dependent dendritic spine remodeling in V1 (Zhou et al., 2020).

Experimental evidence on how the PFC modulates sensory responses in visual cortical areas also arises from studies of attentional mechanisms (Chelazzi, 1995). Extensive research performed in primates revealed that sub-threshold stimulation of the frontal eye field (FEF) in the PFC modulates spatial visual attention (Moore and Armstrong, 2003; Schafer and Moore, 2007; Gregoriou et al., 2009). Experiments in macaque monkeys demonstrated that when two different stimuli are simultaneously presented inside the receptive field of a single neuron, the cell response is modulated by which of the two stimuli is attended (Luck et al., 1997). This is possible because the receptive field of the visual system neuron overlaps with projections of the stimulated FEF area in the PFC (Gilbert and Li, 2013), the FEF being normally subject to attentional mechanisms (Clark and Noudoost, 2014). Accordingly, studies of selective attention have reported increases in local gamma-band and beta-band coherence between PFC and V1 areas (Buschman and Miller, 2007; Gregoriou et al., 2009).

In primates, the oscillatory coupling between the FEF and the visual area V4 seems to be mediated, at least in part, by DA. The antagonism of D1 receptors in the FEF enhances not only the amplitude but also orientation selectivity and reliability of visual responses in V4 (Noudoost and Moore, 2011). Other brain areas also modulate visual cortical regions. Stimulation of the superior colliculus, for instance, contributes to the control of spatial visual attention (Muller et al., 2005). Accordingly, functional magnetic resonance imaging (fMRI) studies of the FEF influence on the visual pathway have shown that sub-threshold FEF stimulation increases the activation of retinotopically corresponding regions in visual areas (Ekstrom et al., 2008). Interestingly, FEF sub-threshold stimuli modulate contrast sensitivity in multiple visual regions in primates (Ekstrom et al., 2009). Experimental observations in rodents similarly indicate a role for the mPFC in attentional processes (Birrell and Brown, 2000). Altogether, these findings raise the possibility that mPFC top-down signals modulate properties of neural circuitries in V1. This is particularly important when planning therapeutic strategies for abnormal physiological conditions of the visual system.

### PFC Signaling, Environmental Enrichment, and Visual Impairments

The etiology of eye conditions that lead to visual impairments are multifactorial and include aging, genetics, infections, and lifestyle^1^. Common disorders that affect visual functions include macular degeneration, retinitis pigmentosa, diabetic retinopathy, glaucoma, retinopathy of prematurity, cataracts, refractive errors, infections, and others (GBD 2019 Blindness and Vision Impairment Collaborators; Vision Loss Expert Group of the Global Burden of Disease Study, 2021). Therapeutic interventions for refractive errors, cataracts, and infections are widely available. As to the rest of the pathologies, although current therapeutic strategies still need significant improvements, ongoing efforts in the biomedical community are likely to develop therapeutic strategies for these conditions. Higher-order visual dysfunctions such as cerebral visual impairment (CVI) in children, instead, represent a serious clinical challenge that is difficult to diagnose and manage, as they are cases of “blindness” due to connectivity damage of central visual pathways with no alterations of the eye (Merabet et al., 2017). While neuroimaging studies may assist in identifying afflicted brain areas in this pathology, developing non-invasive multisensory therapeutic strategies to treat CVI in children is in high demand.

A multisensory stimulation approach that activates higher-order association areas such as the mPFC (Watanasriyakul et al., 2019) and influences V1 functions (Sale et al., 2014), is environmental enrichment (EE). This non-invasive environmental strategy has been actually used to treat deficits of vision (Sale et al., 2007). The capability of EE to promote plasticity in the adult brain (Sale et al., 2009) outlines the therapeutic potential of this strategy in pathological conditions where plasticity is compromised. Amblyopia, for instance, is a condition in which vision in one eye is markedly impaired due to an abnormal visual experience during early life (Holmes and Clarke, 2006). Although this pathology can be easily treated by occlusion or penalization therapy during the early stages of development (Li et al., 2019), it is harder to treat it in adulthood, due to a decrease in brain plasticity that occurs with age (Hensch, 2005; Maya-Vetencourt and Origlia, 2012; Maya-Vetencourt and Pizzorusso, 2013). Interestingly, EE induces full recovery of vision in adult amblyopic animals (Sale et al., 2007). Other experimental strategies (He et al., 2007; Maya-Vetencourt et al., 2008; Morishita et al., 2010; Spolidoro et al., 2011; Spatazza et al., 2013) have also been reported. The phenomenon induced by EE is due to a reduction of intracortical inhibition that seems to parallel an increased expression of the neurotrophic factor BDNF (Baroncelli et al., 2010), all this being surprisingly similar to the plasticizing effects induced by both fluoxetine treatment (Maya-Vetencourt et al., 2008) and food restriction (Spolidoro et al., 2011) in the adult brain. Interestingly, these two last strategies trigger epigenetic mechanisms whereby chromatin remodeling up-regulates the expression of plasticity genes (Spolidoro et al., 2011; Maya-Vetencourt et al., 2011, 2012).

Remarkably, imaging studies by fMRI in humans revealed that watching a video while intermittently cycling on a stationary bike for 4 weeks, in parallel to monocular occlusion, restores some degree of spatial acuity and stereopsis in adult amblyopic patients, this effect being preserved after 1 year of training (Lunghi et al., 2018). This is interesting because physical activity and perceptual learning are major components of an enriched environment and each of them independently promotes amblyopia recovery in adult animals (Baroncelli et al., 2012). In light of this, experimental research using EE in animal models of blindness may provide precious insights into the treatment of higher-order visual dysfunctions such as CVI in children. Other attention-related tasks are likely to be important for the clinical treatment of this pathology.

## Discussion

Understanding how the functional interaction between different brain regions occurs through multisensory integration is a leading edge and clinically relevant area in the neuroscience field, which can be translated into novel and original therapeutic approaches to treat a wide variety of neurological disorders. CVI, for instance, is a rising public health issue with an enormous social and economic impact. Unfortunately, it is one of the most common causes of visual impairment in children, is difficult to diagnose, and has no effective clinical treatment (Afshari et al., 2001; Good et al., 2001).

In line with experimental findings in animal models, diverse studies in humans have shown that practicing perceptual learning promotes amblyopia recovery in adulthood (Levi and Li, 2009; Li et al., 2011). Although more accurate analysis on PFC function and how it may impact sensory functions and cognitive processes are still needed, these findings suggest that DA-driven PFC signaling might underlie, at least in part, some of the effects induced by perceptual learning on V1 areas. Interestingly, the antagonism of D1 receptors in areas of the PFC that regulate attention enhances visual perception (Noudoost and Moore, 2011) whereas EE non-invasively reduces the density of D1 receptors in the PFC of adult animals (Del Arco et al., 2007).

**Figure 2:**
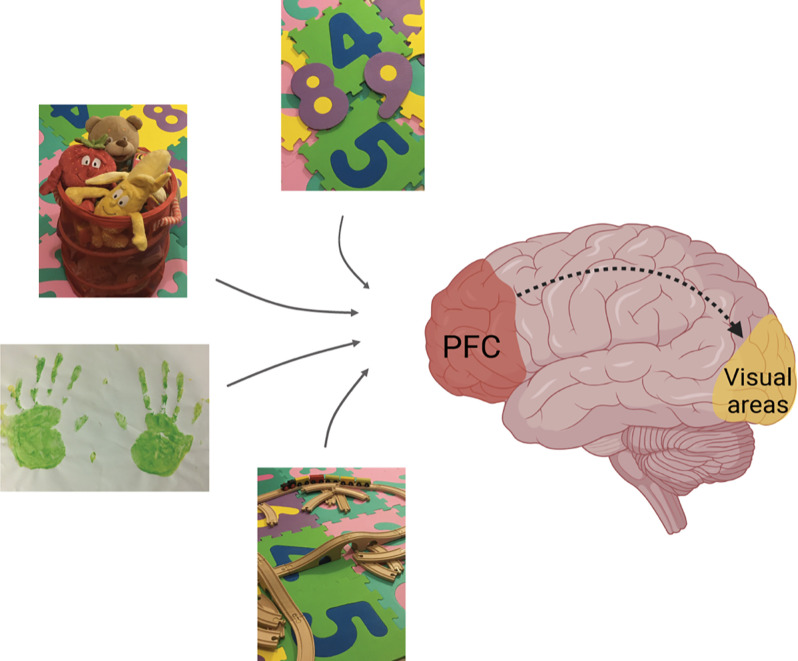
Role of the PFC-V1 interaction in the treatment of visual dysfunctions. Higher-order associative brain areas such as the PFC, which receive inputs from different sensory modalities, seem to modulate information processing at the level of primary sensory systems *via* down-stream signaling (dashed line). We propose that non-invasive multisensory activities and/or attention-related tasks that presumably activate association cortices in the human brain might be beneficial in the treatment of higher-order visual dysfunctions such as CVI in children. These activities include: playing with different inanimate objects, numbers, toy train sets, drawing, and/or other creative activities. Figure created with BioRender.com.

May long-term mPFC stimulation be relevant to the clinical treatment of higher-order visual dysfunctions? Electrophysiological (Golmayo et al., 2003), immunohistochemical (Nguyen et al., 2015), and anatomical (Balog et al., 2019) evidence support this notion. Neuroimaging approaches revealed that abnormalities of synaptic connectivity at central level are a hallmark of these visual pathologies (Merabet et al., 2017). Interestingly, imaging studies in humans have shown that attention-induced mechanisms influence neuronal networks connectivity in diverse visual areas (Silver et al., 2007; Lauritzen et al., 2009). Hence, experimental approaches using non-invasive long-term strategies that promote mPFC signaling and synaptic connectivity, such as perceptual learning and/or attention-related tasks, might be valuable for designing an effective treatment for such disorders (Figure 2). The recent observation that the interaction between auditory and visual stimuli in a behaviorally relevant context modifies visual perception in mice (Garner and Keller, 2021) supports our hypothesis. It is well established that plastic phenomena in the cortex are actively constrained by the gradual appearance of cellular and molecular factors that occur over development (Hensch et al., 1998; Huang et al., 1999; Pizzorusso et al., 2002; McGee et al., 2005; Syken et al., 2006; Putignano et al., 2007; Harauzov et al., 2010; Morishita et al., 2010; Beurdeley et al., 2012; Miyata et al., 2012; Spatazza et al., 2013; Tiraboschi et al., 2013; Apulei et al., 2019; Napoli et al., 2020). It will be interesting to evaluate whether the impact of long-term mPFC signaling on the rescue of visual functions in CVI correlates with variations of the above-mentioned factors. The use of optogenetics may shed light on this issue (Eleftheriou et al., 2017) contributing to the future development of original therapeutic strategies to fight blindness. Transcranial magnetic stimulation (Lefaucheur, 2019) could also be a procedure that may accompany future treatments of higher-order visual impairments. The multisensory and non-invasive nature of EE as a stimulation approach opens new potentialities in the field of higher-order visual dysfunctions where the application of this paradigm, alone or in combination with attentional tasks, might arise as a therapeutic strategy for CVI.

## Author Contributions

All authors discussed the manuscript topics and participated in writing of the review. All authors contributed to the article and approved the submitted version.

## Conflict of Interest

The authors declare that the research was conducted in the absence of any commercial or financial relationships that could be construed as a potential conflict of interest.

## Publisher’s Note

All claims expressed in this article are solely those of the authors and do not necessarily represent those of their affiliated organizations, or those of the publisher, the editors and the reviewers. Any product that may be evaluated in this article, or claim that may be made by its manufacturer, is not guaranteed or endorsed by the publisher.

## Footnotes

^1^WHO. World-report-on-vision. Geneva: World Health Organization, 2021. https://www.iapb.org/learn/resources/the-world-report-on-vision/. Accessed September 15, 2021.
